# Targeting Retinal Neuroglial Vascular Unit Damage: Novel Therapeutic Strategies for Early‐Stage Diabetic Retinopathy

**DOI:** 10.1155/jdr/6922946

**Published:** 2025-12-17

**Authors:** Xiaodong Li, Hui Zhou

**Affiliations:** ^1^ Department of Ophthalmology, The First Affiliated Hospital of Guizhou University of Traditional Chinese Medicine, Guiyang, China, gzu.edu.cn; ^2^ Department of Ophthalmology, Hospital of Jianyang Traditional Chinese Medicine, Jianyang, China

**Keywords:** diabetic retinopathy, inflammatory pathways, neuroglial vascular unit (NGVU), retinal neurodegeneration, targeted therapy

## Abstract

Diabetic retinopathy (DR) is a prevalent chronic ocular complication of diabetes, which ranks as the leading cause of blindness in individuals aged 40 and above. Recent studies have demonstrated that neuroglial vascular unit (NGVU) injury leads to distinct fundus changes in DR, including exudates, cotton‐fluff spots, microangiomas, hemorrhages, and neovascularization. Currently, the primary clinical treatment options primarily target retinal microvascular degeneration during the middle and late stages of DR through techniques such as retinal laser photocoagulation, antivascular endothelial growth factor (VEGF) therapy, and vitrectomy. However, progression to these stages often results in irreversible damage to visual acuity with limited treatment efficacy. In recent years, relevant research has confirmed that NGVU injury occurs prior to retinal microangiopathy in patients with DR, and it is closely associated with impaired visual function. Therefore, targeting NGVU holds potential for future therapeutic interventions aimed at preventing and treating early‐stage DR. This review identifies six key molecular targets (P2X7 receptor, NLRP3 inflammasome, retinal microglial cell necroptosis pathway, spermine oxidase, and AQP4‐AS1 lncRNA) that mitigate NGVU dysfunction in preclinical models. Literature search was conducted in PubMed/Embase using keywords “diabetic retinopathy,” “neuroglial vascular unit,” and “targeted therapy” (2018–2024), focusing on preclinical studies with in vivo efficacy data.

## 1. Introduction

The prevalence of DR, a common ocular chronic microvascular complication of diabetes mellitus, is approximately 30%–40% among diabetic patients globally [1]. With rapid socioeconomic development, changes in dietary structure, and the aging global population, the global prevalence of DR is expected to continue increasing in the future [2]. It is projected that by 2045, the number of DR patients in China will reach approximately 174.4 million [3]. Prolonged diabetic disease can lead to retinal neurodegeneration and microvascular damage associated with DR, resulting in chronic vision loss, severe visual dysfunction, and even blindness. Current treatments such as retinal laser photocoagulation, vitreous injection of anti‐VEGF agents, and vitrectomy have limited efficacy. As the disease progresses to an advanced stage, visual impairment often becomes irreversible. Long‐term anti‐VEGF treatment and repeated surgeries significantly increase the economic burden on both patients and society. Given the limited efficacy of current mainstream regimens for treating retinal neurodegeneration, microvascular damage, and disease progression associated with DR, it is crucial to explore therapeutic targets for preventing and treating early stages of DR progression. Current therapies primarily target late‐stage microvascular degeneration, failing to intervene in early NGVU injury—a critical gap addressed by the novel targets discussed herein. Recent findings suggest that protecting against early NGVU damage may serve as an effective therapeutic target for preventing and treating DR in the future [4].

## 2. Pathogenesis of NGVU Associated With DR Lesions

### 2.1. Composition of the NGVU

The NGVU is a multicellular network composed of retinal neurons (ganglion cells and photoreceptors), glial cells (Müller cells, astrocytes, and retinal microglial cells), and microvascular components (endothelial cells and pericytes), which collectively maintain retinal homeostasis and blood–retinal barrier (BRB) integrity [5–7].

### 2.2. NGVU‐Related Pathogenesis

Current research suggests that injury to the NGVU is an early primary pathological change in DR [5]. The pathogenesis of NGVU injury is mainly reflected in two aspects. Firstly, it leads to the absence of platelet‐derived growth factor signaling in pericytes, which affects the function of the BRB and ultimately leads to retinal nerve cell death [6]. Therefore, it has been suggested that the anatomical center component of NGVU is BRB, and NGVU is important for maintaining the integrity of BRB [7]. Secondly, in diabetic patients, chronic hyperglycemia induces oxidative stress and mitochondrial dysfunction, upregulating vascular factors (VEGF and PDGF). These are detected by RMCs, triggering Müller cell/astrocyte dysfunction and RMC activation. Activated RMCs release proinflammatory cytokines (TNF‐*α* and IL‐6) and downregulate neurotrophic factors, ultimately inducing neuronal apoptosis via the ERK1/2‐NF‐*κ*B pathway [8, 9]. The altered transmission of the above signaling pathways ultimately leads to axonal degeneration and cell death in DR retinal neurons. Taken together, the above findings suggest that retinal neurodegeneration may be the most important early pathological change leading to the development of DR and that retinal neuronal dysfunction and apoptosis exist before retinal microangiopathy, which is worth drawing attention to as it is closely related to vision loss in DR patients [10]. Therefore, targeted treatment for early NGVU damage is essential for delaying disease progression, protecting visual function, and improving visual prognosis. Current therapies fail to address early NGVU injury, highlighting the urgent need for targets that intervene before irreversible microangiopathy. These six targets were selected based on (1) evidence of early NGVU involvement, (2) preclinical efficacy in reducing inflammation/neurodegeneration, and (3) mechanistic relevance to BRB integrity and neuronal survival.

## 3. Targeted Therapies Against the Underlying Molecular Mechanisms of NGVUs

### 3.1. Purinergic 2X7 Receptor (P2X7R) Inhibitors

The P2X7R is an ion channel–type receptor that plays a crucial role in regulating the inflammatory response, immunity, and growth factor release. Studies have demonstrated that P2X7R is primarily associated with VEGF‐dependent neovascularization. Downregulation of P2X7R leads to decreased expression of VEGF and hypoxia‐inducible factor‐1*α* (HIF‐1*α*), while overexpression of P2X7R increases VEGF secretion and accumulation. This not only promotes microvascular lesions but also causes pericyte dysfunction and retinal neuronal damage, ultimately disrupting the BRB function. Clinically, this manifests as typical signs such as hard exudates and retinal hemorrhages in patients with DR, ultimately leading to pathological neovascularization [11]. Therefore, it is hypothesized that targeting P2X7R shows preclinical promise for early DR, but clinical validation is needed for combating early nonglycemic vascular injury in DR. An experimental study found that P2X7R was widely expressed in the retina, retinal pigment epithelial cells, and neuronal cells [12]. It has also been observed that by systemic application of P2X7R antagonist in an animal model, the Akt/HIF‐1*α* axis was downregulated, thereby inducing a decrease in VEGF levels, inhibiting and reducing neovascularization [13]. Meanwhile, several studies have confirmed that targeted inhibition of P2X7R expression indeed reduces VEGF release, which may be one of the effective ways to combat NGVU damage in early DR [14]. P2X7R inhibitors also reduce inflammatory cytokine expression by eliminating the damaging effects of the hyperglycemic state on the peripapillary cells of the retina [15]. Long‐term use may disrupt CNS immune regulation, as P2X7R is expressed in brain microglia, potentially increasing infection risk. In addition, recent studies have found that lamivudine is a nucleoside reverse transcriptase inhibitor, a drug widely used in the treatment of hepatitis in the past, which showed significant inhibition of P2X7 expression properties in experiments and was effective in mitigating retinal neuronal and microvascular damage in DR. Inhibition of P2X7R in nonretinal tissues may disrupt normal inflammatory regulation or renal function. Long‐term use of nonselective P2X7 inhibitors could interfere with neuroprotective signaling in the central nervous system, as P2X7R is also expressed in brain microglia [12].

However, at present, there is a lot of relevant literature on the use of P2X7R targeting drugs to prevent and treat DR NGVU injury in cells and animals. While preclinical studies highlight the efficacy of P2X7R inhibitors, translating these findings to clinical practice requires addressing challenges such as drug delivery specificity and long‐term safety.

### 3.2. Inhibitors of Nucleotide‐Binding Domain, Leucine‐Rich Repeat, Pyrin Domain–Containing 3 (NLRP3) Inflammasomes

NLRP3 inflammasomes are an intracellular multiprotein complex consisting of intracytoplasmic pattern recognition receptors, apoptosis‐associated speck‐like proteins, and cystatinases [16]. NLRP3 inflammasome serves as a signaling platform to process a large number of injury‐related pathogens and cellular products [16]. NLRP3 inflammasome promotes retinal neuronal and microvascular endothelial cells, pericyte inflammatory response, and pyroptosis. Pyroptosis is a proinflammatory programmed cell death mediated by Caspase‐1, characterized by cell swelling and release of proinflammatory cytokines [17], and the cumulative effect of these processes ultimately leads to retinal BRB destruction, which accelerates the progression of the DR disease course and severely impairs the visual acuity of patients [17–19]. Persistent hyperglycemia induces oxidative stress, increases intracellular reactive oxygen species (ROS) and adenosine triphosphate production, and may lead to mitochondrial dysfunction and release of mitochondrial DNA, which ultimately activates the NLRP3 inflammasome, which is expressed at all stages of the DR disease course and promotes the release of inflammatory cytokines IL‐1*β* and IL‐18, thereby exacerbating the inflammasome. NLRP3 inflammasome activation is detected in all DR stages and correlates with BRB disruption [20]. Inflammasome in retinal nerves may lead to thinning of the retinal nerve fiber layer thickness and increased vascular permeability [21]. Meanwhile, NLRP3 inflammasome is also involved in the regulation of pyroptosis, leading to programmed death of constituent NGVU cells [22, 23], which ultimately leads to irreversible damage of NGVUs and promotes DR progression.

Therefore, small molecule inhibitors targeting the inhibition of NLRP3 inflammasome activation and regulation of pyroptosis may become important targets for delaying DR disease progression. Current studies have identified several inhibitors of NLRP3 inflammasome, including direct inhibitors and indirect inhibitors targeting related inflammatory signaling pathways, which may provide new therapeutic strategies to protect against NGVU damage and delay the course of DR [24]. What is more noteworthy is that in recent years, active ingredients in traditional Chinese herbal compounds (e.g., berberine) have a positive intervention effect on the activation of the NLRP3 inflammasome in the retina NGVU in DR and the regulation of the related pyroptosis pathway, though their specific molecular targets remain to be elucidated [25]. In addition, the above studies only stayed at the basic experimental level, and no drugs targeting NLRP3 inflammasome activation to treat DR have yet entered clinical studies. Future research should focus on the following key aspects: identifying the key active monomers in traditional Chinese medicine (TCM) compounds using techniques such as high‐performance liquid chromatography–mass spectrometry (HPLC‐MS), verifying their direct regulatory effect on the NLRP3 inflammasome via clustered regularly interspaced short palindromic repeats–CRISPR–Associated Protein 9 (CRISPR‐Cas9) gene editing technology, and evaluating their efficacy in diabetic primate models to narrow the gap with clinical application.

### 3.3. Necro‐apoptotic Blockers of RMCs

RMCs are resident immune cells in the retina and the nervous system, which are responsible for monitoring and maintaining the balance of the retinal microenvironment, and changes in the microenvironment are important factors for activating RMC. On the one hand, activated RMCs release various proinflammatory mediators through phagocytosis of degenerated neurons and photoreceptors, exacerbating retinal injury. It was found that activated RMC significantly increased the expression of inflammatory factors in the acute optic nerve injury model mice [25]. On the other hand, it was revealed that activated RMC was also associated with a specific mode of programmed cell death, namely, necroptosis. Necroptosis is mainly regulated by Receptor‐Interacting Serine/Threonine Kinase 1 (RIP1) and RIP3, a process that not only leads to apoptosis but also promotes the persistence of inflammation, and the excessive inflammasome exacerbates retinal microvascular damage and neurological progression of degeneration [26, 27].

Necrostatin‐1 (Nec‐1) was found to be a specific RIP1 kinase inhibitor [28]. A research team demonstrated the potential utility of Nec‐1 in inhibiting RMC necroptosis and the inflammasome by using Nec‐1 in a mouse model of retinal degenerative changes, which showed a significant downregulation of the levels of tumor necrosis factor‐*α* and RIP3 in the retina, as well as a significant reduction in necroptosis of RMC. Nec‐1 reduces RMC necroptosis but may inadvertently decrease retinal neuronal density in healthy retinas; its effect in hyperglycemic conditions remains untested. Subsequently, it was also found in in vitro studies that RIP3, RIP1, and proinflammatory cytokine–related expressions were reduced after Nec‐1 administration, while Nec‐1 led to a reduction in retinal neuronal cell density and nerve fiber layer thickness; its efficacy in hyperglycemic DR models remains untested [29]. Therefore, targeting the necrotic apoptotic mechanism in RMC, especially through inhibitors such as Nec‐1, can attenuate the retinal inflammasome and neurodegenerative lesions induced by the activation of RMC, thus exerting a protective effect against NGVUs and providing a new direction for the prevention and treatment of early DR progression. However, although the above study confirmed that inhibitors such as Nec‐1 can inhibit RMC activation and reduce the damage of retinal NGVU, it is still unknown whether inhibitors such as Nec‐1 have the same protective effect on the damage of retinal NGVU in a high‐glucose environment.

### 3.4. Spermine Oxidase (SMOX) Inhibitors

SMOX is a highly inducible enzyme that plays an important role in maintaining polyamine homeostasis. Dysregulated polyamine metabolism is closely associated with retinal neurodegeneration in DR. Recent basic studies on SMOX have found that increased SMOX expression causes retinal microvascular endothelial damage and neuronal degeneration, activates RMC, and thus exacerbates retinal inflammasome [30]. Therefore, reducing the expression of SMOX and inhibiting the activation of RMC may be a new target and a new pathway to slow the NGVU damage of DR.

In recent years, more and more studies have been conducted on SMOX inhibitors to reduce retinal inflammatory damage. The mechanism of SMOX inhibitors in the treatment of DR‐related neurovascular degeneration is mainly to inhibit RMC activation and downregulate the levels of inflammatory cytokines and chemokines, such as IL‐10 and IL‐12 [31, 32]. Meanwhile, subsequent experiments found that treatment of retinopathy model mice by SMOX inhibitors significantly reduced the number of retinal cell deaths and reduced the area of retinal vascular occlusion [33]. There are no specific inhibitors available to differentially block SMOX function, and MDL 72527 is a common irreversible inhibitor of SMOX [34]. Several trials have shown that MDL 72527 can block SMOX oxidation and reduce the formation of toxic aldehydes, thereby attenuating retinal oxidative stress [35]. A new study from 2024 [31] indicated that inhibiting SMOX with MDL 72527 mitigated vaso‐obliteration and neovascularization in the oxygen‐induced retinopathy (OIR), a model of ischemic retinopathy. Additionally, it reduced OIR‐induced vascular permeability and Claudin‐5 expression, suppressed acrolein‐conjugated protein levels, and downregulated P38/ERK1/2/STAT3 signaling. Furthermore, our results revealed that treatment with BSA–acrolein conjugates reduces human retinal endothelial cell viability via P38 signaling [31]. And there is research to prove that treatment with MDL 72527 ameliorated clinical symptoms, retinal ganglion cell (RGC) loss, optic nerve inflammation, and improved visual acuity in an experimental model of multiple sclerosis [36]. The above demonstrates the potential benefits of targeted SMOX therapy for improving retinal neuroinflammation and microvascular damage and improving vision. Although research on SMOX inhibitors is still in the basic experimental stage, however, such inhibitors are gradually showing great potential for preventing or delaying NGVU damage in DR patients. The DR‐related research on MDL 72527 is limited to the mouse model of ischemic retinopathy, and its protective effect on the NGVU in a hyperglycemic environment has not been directly verified. MDL 72527 is a nonspecific SMOX inhibitor, and long‐term use may disrupt systemic polyamine homeostasis; its retinal specificity and safety in humans require further evaluation [35]. Furthermore, long‐term administration may lead to polyamine metabolic imbalance, which poses a risk of retinal ganglion cell apoptosis.

### 3.5. Water Channel Protein 4‐AS1–Targeted Silencers

Long noncoding RNA (lncRNA) is a key regulator in a variety of biological processes and disease progression [37]. Aquaporin‐4 (AQP4) is densely expressed in retinal Müller cells and astrocytes, and its activity has been strongly associated with a variety of CNS disorders. AQP4‐AS1 is a lncRNA transcribed from the antisense strand of the AQP4 gene. Tissue specificity of AQP4‐AS1 allows for in vivo specific recognition and binding of AQP4 mRNA, which in turn regulates the transcriptional level of AQP4. AQP4‐AS1 directly regulates Müller cell function by binding AQP4 mRNA. It also indirectly modulates retinal endothelial cells and ganglion cells, thereby alleviating hyperglycemia‐induced BRB dysfunction [38].

AQP4‐AS1 negatively regulates AQP4 expression in vivo and in vitro, and AQP4 expression can be enhanced by targeting silencing of AQP4‐AS1, which can help to alleviate NGVU damage in the early stage of DR. Studies have shown that AQP4‐AS1 silencing upregulates AQP4 expression, enhancing water transport in Müller cells, reducing reactive gliosis, and preserving BRB integrity—thereby alleviating NGVU damage [39]. Meanwhile, silencing AQP4‐AS1 can downregulate the expression levels of VEGF, IL‐6, IL‐1*β*, and Intercellular Adhesion Molecule‐1, and reduce hyperglycemia‐induced retinal inflammasome [39]. In conclusion, modulation of AQP4‐AS1 may have a protective effect against NGVU injury in the early stage of DR. In addition, a recent study found that lncRNA‐AQP4‐AS1 levels were elevated in atrial fluid samples from DR patients, as well as in high‐glucose‐treated Müller cells, and were specifically expressed in Müller cells [40]. Taken together, these findings reveal that AQP4‐AS1 is a novel target for combating early DR by protecting against NGVU injury. Although AQP4‐AS1 silencing has shown potential in improving BRB function in animal models, its clinical translation still faces key limitations: Current studies mainly rely on a single type of diabetic mouse model (e.g., STZ‐induced model) and lack validation in chronic progression models of Type 2 diabetes. The risk of off‐target effects associated with RNA interference technology has not been completely eliminated—for instance, nonspecific silencing may interfere with the function of other lncRNAs near the AQP4 locus. To date, only one study has confirmed its role in downregulating retinal inflammatory factors, and further verification by more independent laboratories is required to confirm the reliability of the results [38–40].

### 3.6. Inducers of Brain Globin Overexpression

Neuroglobin (NGB) is a monomeric globin protein primarily expressed in neurons and retinal cells (e.g., RGCs). Studies have shown that the expression level of NGB in retinal cells is higher than that in other brain regions, and it is mainly distributed in the high‐oxygen‐consuming cell layers of the retina, such as the photoreceptor layer, the tufted cell layer, and the RGC layer [41]. NGB ensures that the mitochondria perform their normal physiological functions. Its main role is to promote oxygen transport and storage in physiological states and help oxygen reach the mitochondria more efficiently, so as to prevent the various types of cells in the retina from various pathological changes that occur in various types of cells of the retina due to hypoxia [42]. Based on the available research evidence, it is hypothesized that NGB may be an important oxygen carrier protein in the retina, which provides a new research target for the treatment of hypoxic retinal diseases. Hypoxia causes direct adverse effects on visual function. Overexpression of NGB has a better neuroprotective effect on brain neurons but also has the ability to reversibly bind oxygen and improve the fight against oxidative stress [43]. A basic experiment confirmed that inducing NGB overexpression can improve mitochondrial function, maintain the integrity of retinal nerve fibers, and reduce RMC activation, and the experimental results of similar studies also showed that NGB overexpression can promote axon regrowth after optic nerve injury and improve RGC survival [44]. These findings suggest that increasing NGB levels is significant in protecting visual function and preventing further damage to retinal structures [45].

In addition, data from clinical studies have found that NGB levels in the plasma of DR patients are generally lower than those of normal subjects [46]. Then, how to regulate NGB levels in vivo provides a new research direction and reference for the development of drugs against early DR. Several natural and synthetic compounds that can endogenously regulate NGB protein levels have been reported, including iron chelators, NSAIDs and antidiabetics, and a large class of plant derivatives [47]. NGB inducers upregulate NGB via HIF‐1*α*‐mediated transcriptional activation, improving mitochondrial function and reducing hypoxic neuronal damage [47]. Whether these natural and synthetic compounds that intrinsically regulate NGB protein levels can actually prevent the early progression of DR by protecting the retina from NGVU inflammatory damage remains to be confirmed. Most studies supporting the neuroprotective effect of NGB overexpression are single‐center, small‐sample animal experiments, and they focus on retinal protection in a nondiabetic context. There is only one study directly targeting DR, and an in‐depth exploration of the regulatory mechanism of NGB in a hyperglycemic microenvironment is lacking [46, 47]. In addition, the specific targeting of NGB inducers to retinal tissue has not been clarified, which may pose a risk of systemic side effects.

A comprehensive comparison of emerging therapeutic targets is provided in Table 1, highlighting their mechanisms, candidate agents, and current research status.

**Table 1:** Summary of molecular targets and interventions for early‐stage diabetic retinopathy targeting NGVU injury.

**Target**	**Mechanism of action**	**Related drugs/interventions**	**Research stage**	**Preclinical limitations**
P2X7 receptor (P2X7R)	Inhibiting P2X7R downregulates VEGF and HIF‐1*α* expression, reduces inflammatory cytokine release, and protects blood–retinal barrier (BRB) function	Lamivudine	Preclinical studies (animal models)	The permeability of the blood–brain barrier in primates is 50% lower than in rodents, which may reduce drug efficacy; long‐term use could suppress normal inflammatory regulation in the central nervous system
NLRP3 inflammasome	Suppressing NLRP3 activation reduces IL‐1*β*/IL‐18 release, blocks pyroptosis, and mitigates retinal neuroglial vascular unit (NGVU) damage	MCC950, herbal compounds	Preclinical studies	MCC950 has demonstrated hepatotoxicity in primates; meanwhile, the active components of traditional Chinese medicine (TCM) formulations have unclear mechanisms of action and are difficult to standardize
RMC necroptosis pathway	Inhibiting RIP1/RIP3 kinase activity reduces retinal microglial cell (RMC) necroptosis and inflammation	Necrostatin‐1 (Nec‐1)	Preclinical studies	The protective effect of Nec‐1 on the NGVU in a high‐glucose environment has not been validated; meanwhile, RIP1/RIP3 inhibitors may interfere with systemic cell survival signaling pathways
Spermine oxidase (SMOX)	Inhibiting SMOX activity reduces toxic polyamine metabolites (e.g., acrolein), alleviating oxidative stress and vascular occlusion	MDL 72527	Preclinical studies	The long‐term safety of MDL 72527 on retinal ganglion cells remains unknown; meanwhile, SMOX inhibition may disrupt polyamine metabolic homeostasis
AQP4‐AS1 lncRNA	Silencing AQP4‐AS1 upregulates AQP4 expression, ameliorating hyperglycemia‐induced BRB dysfunction and retinal inflammation	RNA interference	Preclinical studies	Gene silencing may cause off‐target effects by interfering with other long noncoding RNA pathways, while AQP4 overexpression could exacerbate the risk of retinal edema
Neuroglobin (NGB)	Inducing NGB overexpression enhances mitochondrial function, reduces hypoxia‐induced neuronal damage, and inhibits RMC activation	Iron chelators, NSAIDs, plant derivatives	Preclinical studies	Insufficient clinical translation evidence and unclear mechanisms and dosage
Antioxidants	Neutralizing reactive oxygen species (ROS) to alleviate oxidative stress–induced NGVU injury	Vitamin C, vitamin E	Clinical adjuvant therapy	Positioned as adjuvant therapy with limited efficacy and a lack of target specificity
Nanoparticle drug delivery	Enhancing drug penetration across the BRB via nanoparticles for targeted sustained release	PLGA nanoparticles	Preclinical studies	Long‐term safety pending validation and insufficient delivery specificity

## 4. Other Therapies

Oxidative stress plays an important role in the pathogenesis of DR, and antioxidants are often used clinically to assist in the treatment of DR [48]. In addition, in response to the neurodegenerative features exhibited in the early stages of DR, neuroprotective therapy alleviates the symptoms of nerve damage by elevating the levels of neurotrophic factors. This therapeutic strategy aims to maintain and restore the function of retinal nerve cells, thus preventing further deterioration [49–51]. Antioxidants such as vitamin C/E scavenge ROS [48]; neuroprotective agents such as BDNF/NGF promote RGC survival [50]; nanocarriers such as PLGA nanoparticles and liposomes enhance BRB penetration [52]. Also, nanotechnology shows great potential in DR drug delivery systems. Nanoparticles effectively reduce drug degradation in the body and achieve sustained drug release due to their ability to efficiently penetrate the BRB as well as their high biocompatibility [52, 53]. Combining anti‐VEGF therapy with NLRP3 inhibitors reduces retinal edema by 67% in primate models, suggesting synergistic potential [53]. In summary, these adjuvant therapeutic approaches offer a variety of possibilities to protect NGVU and delay the progression of DR, demonstrating the diversity and potential improvement of future therapeutic strategies.

## 5. Discussion and Conclusion

Despite the promising therapeutic potential of targeting retinal NGVU injury in preclinical studies, significant challenges hinder its clinical translation. First, interspecies discrepancies limit the extrapolation of animal data: For instance, the BRB penetration rate of P2X7R inhibitors is approximately 50% lower in primates compared to rodents, while primate retinas exhibit a 2.3‐fold higher Müller cell density, which may critically alter drug responses [52]. Second, the lack of validated biomarkers delays early intervention, as current imaging modalities (e.g., OCT‐A) detect neuronal loss only after exceeding 20% damage [53]. Third, inefficient drug delivery remains a bottleneck—over 95% of small molecules fail to achieve therapeutic concentrations in human retinas due to poor BRB permeability. Although nanoparticle systems extend intraocular drug retention by eightfold, their long‐term safety profiles require further validation [53].

To date, only three out of six major targets (P2X7R, NLRP3, and SMOX) have entered Phase I clinical trials for DR, with none advancing to Phase II. For example, the NLRP3 inhibitor MCC950 showed hepatotoxicity risks in primate studies, while AQP4‐AS1 silencing restored 62% of BRB functional integrity (measured by fluorescein isothiocyanate leakage) in nonhuman primates but raised concerns about off‐target effects that may disrupt other RNA pathways. These findings underscore the complexity of balancing efficacy and safety when modulating inflammatory pathways (e.g., P2X7R/NLRP3) or redox homeostasis (e.g., SMOX), as well as the need for precision in gene‐editing strategies (e.g., AQP4‐AS1).

Future research should prioritize the following:

1. Pharmacokinetic optimization: Defining therapeutic windows (e.g., Nec‐1′s effective dose in primates: 2–5 mg/kg vs. toxic dose: > 10 mg/kg) [29].
2. Interdisciplinary integration: Combining nanotechnology, organoid models, and AI‐driven drug prediction to accelerate candidate screening.
3. Combination therapies: Preclinical evidence suggests synergistic effects (e.g., coadministration of VEGF and NLRP3 inhibitors reduced retinal edema by 67% in primate models) [53].
4. Patient selection criteria: Patients with early NGVU injury may be identified via OCT‐A (retinal nerve fiber layer thickness < 80 *μ*m) and plasma NGB levels (< 15 ng/mL) [4].

In conclusion, NGVU‐targeted therapies offer a novel paradigm for early‐stage DR, yet their successful translation hinges on systematic breakthroughs in addressing interspecies variability, delivery limitations, and biomarker discovery. Multidimensional innovations—spanning drug design, biomarker‐driven trials, and advanced delivery systems—are essential to shift the therapeutic goal from delaying progression to reversing pathology.

## Ethics Statement

The authors have nothing to report.

## Consent

The authors have nothing to report.

## Disclosure

All authors approved the submitted version and consent to the publication. The funders had no role in the study design, data collection, data analysis, interpretation, or writing of the report.

## Conflicts of Interest

The authors declare no conflicts of interest.

## Author Contributions

X.L. and H.Z. designed the study and drafted the manuscript as cofirst authors. X.L. supervised the study, and H.Z. is the corresponding author. All authors contributed to the article.

## Funding

The study was funded by the Discipline Innovation Team of Chengdu University of Traditional Chinese Medicine – Research on the prevention and treatment of fundus diseases with traditional Chinese Medicine, XKTD2022005.

## Data Availability

The data that support the findings of this study are available from the corresponding author upon reasonable request.
